# Integrative analysis of multiple diverse omics datasets by sparse group multitask regression

**DOI:** 10.3389/fcell.2014.00062

**Published:** 2014-10-27

**Authors:** Dongdong Lin, Jigang Zhang, Jingyao Li, Hao He, Hong-Wen Deng, Yu-Ping Wang

**Affiliations:** ^1^Biomedical Engineering Department, Tulane UniversityNew Orleans, LA, USA; ^2^Center for Bioinformatics and Genomics, Tulane UniversityNew Orleans, LA, USA; ^3^Department of Biostatistics and Bioinformatics, Tulane UniversityNew Orleans, LA, USA

**Keywords:** sparse regression, multitask learning, group lasso, significant test, osteoporosis

## Abstract

A variety of high throughput genome-wide assays enable the exploration of genetic risk factors underlying complex traits. Although these studies have remarkable impact on identifying susceptible biomarkers, they suffer from issues such as limited sample size and low reproducibility. Combining individual studies of different genetic levels/platforms has the promise to improve the power and consistency of biomarker identification. In this paper, we propose a novel integrative method, namely sparse group multitask regression, for integrating diverse omics datasets, platforms, and populations to identify risk genes/factors of complex diseases. This method combines multitask learning with sparse group regularization, which will: (1) treat the biomarker identification in each single study as a task and then combine them by multitask learning; (2) group variables from all studies for identifying significant genes; (3) enforce sparse constraint on groups of variables to overcome the “small sample, but large variables” problem. We introduce two sparse group penalties: sparse group lasso and sparse group ridge in our multitask model, and provide an effective algorithm for each model. In addition, we propose a significance test for the identification of potential risk genes. Two simulation studies are performed to evaluate the performance of our integrative method by comparing it with conventional meta-analysis method. The results show that our sparse group multitask method outperforms meta-analysis method significantly. In an application to our osteoporosis studies, 7 genes are identified as significant genes by our method and are found to have significant effects in other three independent studies for validation. The most significant gene SOD2 has been identified in our previous osteoporosis study involving the same expression dataset. Several other genes such as TREML2, HTR1E, and GLO1 are shown to be novel susceptible genes for osteoporosis, as confirmed from other studies.

## Introduction

Increasing amounts of high-throughput biological data have been collected to investigate the genetic mechanism underlying complex traits at different levels, e.g., genomics, transcriptomics, proteomics, and metabolomics. However, there are usually two bottlenecks for these genetic studies. One is availability of limited sample size due to the experimental cost. Small sample size can lead to the loss of detection power and the reduction of confidence on identified biomarkers. To analyze data with small sample size but large variables is still a challenging statistical problem (Hamid et al., 2009). The other is that biomarkers identified from these different studies often suffer from poor reproducibility. This issue could be caused by many factors such as differences on profiling techniques, demographic, and ancestral information of subjects, sample sizes, and quality control in these datasets (Phan et al., 2012; Song et al., 2012). To increase the power and consistency of biomarker identification, integrating the information of diverse biological datasets from different levels and platforms shows great promise and is highly demanded.

Methods for integration of diverse biological datasets include conventional meta-analysis and a variety of integrative approaches recently developed (Huttenhower et al., 2006; Liu et al., 2013). Meta-analysis is a statistical method to summarize the *p*-values or statistics (e.g., z-score) from each individual dataset (Evangelou and Ioannidis, 2013). There are a dozen of approaches for combing multiple *p*-values or statistics such as Fisher method. Meta-analysis is usually used to find common features across multiple datasets with different sample sizes and platforms but under the same hypothesis (Rhodes and Chinnaiyan, 2005). Recently, a number of integrative approaches have been developed, which are based on machine learning and statistical methods (Zhang et al., 2010; Kirk et al., 2012; Xiong et al., 2012). They can analyze multiple datasets from: (1) different platforms and levels but for the same subjects; (2) same platforms but different levels and subjects; (3) different platforms but for the same levels and subjects. They have been successfully used for various applications such as a single or a set of biomarker identification (Chen et al., 2013), gene-gene interaction prediction (Troyanskaya et al., 2003), and genetic network construction (Balbin et al., 2013). The results in these studies demonstrate the advantage of integrating multiple diverse datasets over analyzing them individually.

In this work, we propose a novel method for integrating multiple datasets from different platforms, levels, and samples to identify common biomarkers (e.g., genes). The method was based on multitask regression model enforced with sparse group regularization, which can overcome the “small sample size, but large number of variables” problem. Multitask learning method has been successfully applied to medical imaging data fusion, where multiple types of images (e.g., CT, MRI) were combined for identifying susceptible brain regions and improving disease classification (Zhang and Shen, 2012). Among various sparse regularization terms, the use of sparse group penalty has been shown to outperform other penalties such as lasso in our previous study of pair-wise genomic data integration (Lin et al., 2013). In this study, we enforce two sparse group penalties [i.e., sparse group lasso (Friedman et al., 2010) and sparse group ridge (Chen et al., 2010a)] into the multitask regression model for data integration. We assume a regression model for each dataset as a task, and then multiple regression models will be considered as multiple tasks. Variables from all datasets will be grouped by specific units (e.g., genes). A sparse group penalty is introduced with the aims to (1) reduce dimensionality, i.e., removing a number of irrelevant genes; (2) perform group-wise feature selection, i.e., removing SNPs or expression measurements from the same gene. An effective algorithm based on alternative direction method (ADM) is proposed to solve the model. Based on the estimation of the model, a statistical test is constructed for the identification of potentially causal genes. We perform two simulation studies with both fixed and dynamic genetic effects to evaluate our sparse regression methods, which shows that our sparse group multitask regression model can increase the power of detecting risk genes by integrating multiple diverse datasets effectively. Real data analysis on four osteoporosis studies identifies some significant genes with highly susceptible to bone mineral density and osteoporosis.

## Materials and methods

In this section, we will first introduce the sparse group multitask regression model and then propose an effective algorithm based on ADM to solve the model. Finally, a gene based statistical test is constructed to give the level of significance for each selected gene.

### Sparse group multitask regression model

We assume *T* independent datasets collected from *K* levels of genomic data (e.g., SNP, mRNA) with *P*_*k*_(*k* = 1, …, *K*) platforms (e.g., Affymetrix, Illumina) for each level, and thus $T\;=\sum_{k\;=\;1}^{K}P_{k}$. The number of observations in each dataset is denoted by *n*_*i*_, *i* = 1, … *T*. Sample sizes could also be different due to the diversity of protocols in each experiment. The measurement matrix of each experiment is denoted by *X*^(*i*)^ ∈ *R*^*n*_*i*_ × *d*_*i*_^, *i* = 1, …, *T*, where d_i_ is the dimension of features in the *i*-th dataset, and usually *d*_*i*_ >> *n*_*i*_. These features (e.g., SNPs and mRNA expression probes) are annotated to the genes and we assume that the genes in different datasets are the same, denoted by *G* = {*G*_*i*_|*i* = 1, … *Q*}. For example, all SNPs and mRNA expressions are tested for the same set of genes *G*. To reduce scale differences among different levels and platforms, the features in *X*^(*i*)^s will be normalized to have zero mean and unit standard deviation. The phenotypic response in each dataset is *Y*^(*i*)^ ∈ *R*^*n*_*i*_^, *i* = 1, … *T*, which can be binary or quantitative trait. The study is to identify biomarkers shared by different experiments for the same phenotype. The coefficient matrix for the regression model is denoted by $C={\left[C^{{\left(1\right)}^{\prime }},C^{{\left(2\right)}^{\prime }},\ldots ,C^{{\left(T\right)}^{\prime }}\right]}^{\prime }$, where *C*^(*i*)^ ∈ *R*^*d*_*i*_^ is the coefficient vector of the *i*-th model *Link* (*Y*^(*i*)^) = *X*^(*i*)^*C*^(*i*)^, and *Link*(.) is the known link function.

Multitask learning is adopted in this study for identifying the shared biomarkers across a set of distinct but correlated tasks for better accuracy. In this context, each regression model for an experiment under different level and/or platform is considered as a task. For the sake of simplicity, we assume a linear regression model for each experiment with quantitative trait (i.e., link function will be the identity matrix). The loss function for each model *L*^(*i*)^ (*X*^(*i*)^, *C*^(*i*)^) can be derived from the negative log likelihood function and thus the total loss function for the multitask regression model is $L(X,C)=\sum_{i\;=\;1}^{T}L^{\left(i\right)}\left(X^{\left(i\right)},\;C^{\left(i\right)}\right)$.

Many conventional regression methods become ineffective for processing the large scale biological data, which usually have small sample sizes and large number of features. This issue can be addressed by introducing sparse penalty in the model. We propose a sparse multitask regression model as follows:

(1) $$min_{C}L(X,C)+\Phi (C)$$

where Φ(***C***) is the sparse penalty function. Two popular penalties are used: sparse group lasso and sparse group ridge, and the corresponding models are denoted by multitask-sglasso and multitask-sgridge, respectively. For multitask-sglasso, $\Phi (C)=\lambda_{1}\sum_{q\;=\;1}^{Q}{\left\|C_{\left\{k\;\in \;G_{q}\right\}}\right\|}_{2}+\lambda_{2}{\left\|C\right\|}_{1,\;1}$, where ***C***_{*k* ∈ *G*_*q*_}_ indicates a subset of vector ***C*** corresponding to the set of features annotated to gene *G*_*q*_ from *T*types of datasets and ${\left\|C\right\|}_{1,\;1}=\sum_{i\;=\;1}^{T}\sum_{k\;=\;1}^{d_{i}}\left|C^{\left(i,k\right)}\right|$ is the *l*-1 norm on ***C***. This sparse group lasso penalty groups features from all datasets based on genes to perform gene level selection. The *l*-1 norm penalty on ***C*** can further remove those irrelevant features from each gene. This bi-level feature selection penalty has been proven to outperform several other single level sparse penalties such as lasso, group lasso, and elastic net for feature identification. For multitask-sgridge, a composite sparse penalty, i.e., group ridge penalty $\Phi (C)={\sum_{q\;=\;1}^{Q}\left\|C_{\left\{k\;\in \;G_{q}\right\}}\right\|}_{1}^{2}$, is imposed on ***C*** to perform bi-level feature selection, where the features are also grouped by genes. The penalty uses the inner *l*-1 norm penalty on ***C***_{*k* ∈ *G*_*q*_}_ to achieve the sparsity within each gene while the outer ridge penalty to perform ridge regression at the gene level. This group ridge penalty has also been found to give higher power in identifying causal genes in high dimensional genomic dataset than other single level sparse penalties (Chen et al., 2010a).

In this study, we adopt these two bi-level penalties in our multitask regression models to integrate multiple diverse genomic datasets for gene-based test. Specifically, these two sparse group multitask regression models are formulated as follows:

(2) $$\begin{array}{l} \text{Multitask-sglasso: }min_{C}\sum_{i\;=\;1}^{K}\omega_{i}\sum_{j\;=\;1}^{P_{i}}\delta_{j}{\left\|Y^{\left(i,j\right)}-X^{\left(i,j\right)}C^{\left(i,j\right)}\right\|}_{F}^{2}\\ \;+\lambda_{1}\sum_{q\;=\;1}^{Q}{\left\|C_{\left\{k\;\in \;G_{q}\right\}}\right\|}_{2}+\lambda_{2}{\left\|C\right\|}_{1,\;1} \end{array}$$

(3) $$\begin{array}{l} \text{Multitask-sgridge: }min_{C}\sum_{i\;=\;1}^{K}\omega_{i}\sum_{j\;=\;1}^{P_{i}}\delta_{j}{\left\|Y^{\left(i,j\right)}-X^{\left(i,j\right)}C^{\left(i,j\right)}\right\|}_{F}^{2}\\ \;+\lambda \sum_{q\;=\;1}^{Q}{\left\|C_{\left\{k\;\in \;G_{q}\right\}}\right\|}_{1}^{2} \end{array}$$

where ω_*i*_s are the weights for the loss function of different levels of datasets, and δ_*j*_s are the weights accounting for the sample size differences among the experiments of the same type of datasets. To be more specific, ω_*i*_s reflect the prior knowledge on the importance of different levels of measurements, e.g., SNP, gene expression, and proteomics. We choose ω_*i*_ = 1, *i* = 1, 2, *l* … *K* in this work, assuming that all levels of measurements contain the same important genetic information. Larger sample size is expected to provide more reliable significance test on biomarkers; therefore, the weight for the experiment under the *j*-th platform to measure the *i*-th level of genomic data is given by $\delta_{j}=\frac{n_{j}}{\sum_{j\;=\;1}^{P_{i}}n_{j}},j\in P_{i}$, where λ_1_, λ_2_, and λ are the tuning parameters to control the sparsity of genes and the number of features in the models.

It could be noted that our sparse multitask regression model can be taken as the generalization of those existing sparse regression models to the representation of multiple datasets from different levels and/or platforms. For example, when *K* = 1, *P* = 1, it is sparse regression model for single dataset as used in Chen et al. (2010a) and Simon et al. (2013); when *K* = 1, *P* > 1, it can be reduced to sparse model on multiple datasets at the same level but from different platforms, similar to the work in Ma et al. (2011); when *K* > 1, *P* = 1, it can work for multiple datasets at different levels.

### Solution algorithm by alternative direct method (ADM)

Although both (2) and (3) are convex optimization problem with global solutions, the non-smoothness and the composite norms still cause difficulties in solving the optimization. Several algorithms have been studied to address such an issue for single task regression models, e.g., second-order cone programming (SOCP) algorithm (Candes and Romberg, 2005), spectral projected gradient method (SPGL1) (van den Berg et al., 2008), accelerated gradient method (SLEP) (Liu et al., 2009), block-coordinate descent algorithm and SpaRSA (Wright et al., 2009). In sparse multitask regression model, since the loss function is separable, these algorithms are still applicable but expensive in computations. In this study, we apply ADM to solve sparse multitask regression model. ADM uses the splitting strategy to decompose the optimization problem into several easily solvable ones and updates the variable in each subproblem iteratively until the convergence is achieved. It has been successfully applied to solve many convex or non-convex optimization problems, such as lasso (Yang and Zhang, 2011), total variation regularization (Esser, 2009), matrix decomposition and our recent work on sparse low rank decomposition (Dongdong et al., 2013). Deng et al. compared ADM with several other algorithms and found that ADM outperformed others with more robustness and faster computation (Deng et al., 2013).

Taking the model in (2) for example, we use ADM to split the penalties and transform (2) into the following optimization:

(4) $$\begin{array}{l} min_{C}\sum_{i\;=\;1}^{K}\omega_{i}\sum_{j\;=\;1}^{P_{i}}\delta_{j}{\left\|Y^{\left(i,j\right)}-X^{\left(i,j\right)}C^{\left(i,j\right)}\right\|}_{F}^{2}+\lambda_{1}\sum_{q\;=\;1}^{Q}{\left\|V_{1}{}_{\left\{k\;\in \;G_{q}\right\}}\right\|}_{2}\\ \;+\lambda_{2}{\left\|V_{2}\right\|}_{1,1}\\ \;s.t.\;C=V_{1},C=V_{2} \end{array}$$

where ***V*_1_**, ***V*_2_** are two variables making the loss function separable. The augmented Lagrange function can be derived as

(5) $$\begin{array}{l} L\left(C,V_{1},V_{2},D_{1},D_{2},\lambda_{1},\lambda_{2},\mu ,\rho \right)\\ =\;\sum_{i\;=\;1}^{K}\omega_{i}\sum_{j\;=\;1}^{P_{i}}\delta_{j}{\left\|Y^{\left(i,j\right)}-X^{\left(i,j\right)}C^{\left(i,j\right)}\right\|}_{F}^{2}+\lambda_{1}\sum_{q\;=\;1}^{Q}{\left\|V_{1}{}_{\left\{k\;\in \;G_{q}\right\}}\right\|}_{2}\\ +\lambda_{2}{\left\|V_{2}\right\|}_{1,1}+\frac{\rho }{2}{\left\|C-V_{1}-D_{1}\right\|}_{2}^{2}+\frac{\rho }{2}{\left\|C-V_{2}-D_{2}\right\|}_{2}^{2} \end{array}$$

where ρ is augmentedLagrangian parameter which can be updated iteratively; ***D*_1_**, ***D*_2_** are the Lagrange multipliers to approximate the residuals between ***C*** and ***V*_1_**, ***V*_2_**, respectively. Since the objective function and constraints are both separable and convex, ADM method is effective to solve {***C*, *V_1_*, *V_2_*, *D_1_*, *D*_2_**} sequentially. We present the algorithm for solving multitask-sglasso by ADM in Table 1.

**Table 1:** **Algorithm of solving multitask-sglasso by ADM**.

1	Initialization: *k* = 0, choose λ_1_, λ_2_, μ, ρ, > 0, ***V*^0^_1_, *V*^0^_2_, *D*^0^_1_, *D*^0^_2_**
2	Repeat:
3	***C*^*k* + 1^** ← *argmin*_*A*_*L* (***C*, *V*^k^_1_, *V*^k^_2_, *D*^k^_1_, *D*^k^_2_)**
4	***V*^*k* + 1^_1_** ← *argmin*_***V***_1__L (***C*^*k* + 1^, *V*_1_, *V*^k^_2_, *D*^k^_1_, *D*^k^_2_**)
	= argmin**_*V*_1__** $\frac{\rho }{2}$ ǁ***C*^*k* + 1^ − *V*_1_ − *D*^k^_1_ǁ^2^_2_ + λ_1_** $\sum_{q\;=\;1}^{Q}$ ǁ***V***_**1**{*k* ∈ *G*_*q*}__ǁ_2_
5	***V*^*k* + 1^_2_** ← *argmin***_*V*_2__** *L* (***C*^*k* + 1^, *V*^k + 1^_1_, *V*_2_, *D*^k^_1_, *D*^k^_2_**)
	= *argmin***_*V*_2__** $\frac{\rho }{2}$ ǁ***C*^*k* + 1^ − *V*_2_ − *D*^k^_2_**ǁ^2^_2_ + λ_2_ ǁ***V*_2_**ǁ_1, 1_
6	Update Lagrange multipliers
	***D*^*k* + 1^_1_ ← *D*^k^_1_ − *C*^*k* + 1^ + *V*^*k* + 1^_1_**
	***D*^*k* + 1^_2_ ← *D*^k^_2_ − *C*^*k* + 1^ + *V*^*k* + 1^_2_**
7	Update iteration *k* ← *k* + 1
8	Until some stopping criterion is satisfied

Remark 1. We decouple (2) into several small convex optimization problems. Step 3 is a regular least square estimation on matrix ***C***, where an analytical solution can be derived. Step 4 is a classical sparse group lasso minimization, which can be solved efficiently by block coordinate decent in Sprechmann et al. (2011). Step 5 is a simple lasso problem, which can also be solved by soft-thresholding. The division of complex optimization into several simple sub-optimizations will improve the efficiency of computation.

Remark 2. We adopt the stopping criterion as suggested by Boyd et al. (2010) that both primal res_pri_ and dual res_dual_ residuals must be small, i.e., *res*_*pri*_ ≤ ε_*pri*_, *res*_*dual*_ ≤ ε_*dual*_, where primal residual indicates the difference between ***C*** and ***V*_1_** (***V*_2_**) while dual residual measures the difference between ***V*_1_** (***V*_2_**) and the values at the last iteration.

Remark 3. The convergence rate depends on the choice of Lagrangian parameter ρ. Some studies adjust ρ based on primal and dual variables iteratively to speed up the convergence. In this work, we update ρ by keeping the ratio between primal and dual residual norms within a given interval, say [0.1, 10] until they both converge to zeros.

For optimization (3), it can similarly be transformed into ADM formulation where only one splitting variable (i.e., ***V*_1_**) is needed to separate (3) into two subproblems. The estimation of ***V*_1_** at Step 4 can be replaced by:

(6) $$V_{1}^{k\;+\;1}\text{​}\leftarrow argmin_{V_{1}}\frac{\rho }{2}\text{​}{\left\|C^{k\;+\;1}-V_{1}-D_{1}^{k}\right\|}_{2}^{2}\text{​}+\lambda \sum_{q\;=\;1}^{Q}{\left\|C_{\left\{k\;\in \;G_{q}\right\}}\right\|}_{1}^{2}$$

where soft-threshold can be used to get the solution.

### Statistical test

λ_1_, λ_2_, and λ are tuning parameters used to control the number of genes and features within a gene. The K-fold cross validation is widely used to select optimal values of these parameters. Briefly, the subjects are divided into k groups, where k−1 groups of subjects are used for estimating the coefficient matrix ***C*** and the rest group of subjects is used to calculate the prediction error by the estimated ***C***. We set λ_1_, λ_2_, and λ to [10^0.1^, 10^0.2^, …, 10^3^] with 30 values. We search the 30 × 30 grid to find an optimal combination of (λ^*^_1_, λ^*^_2_) for multitask-sglasso and similarly optimal value of λ^*^ for multitask-sgridge by 5-fold cross validation. Finally, the estimate of ***C*** can be calculated by the derived optimal parameters.

To test the significance of identified biomarkers with non-zeros coefficients at ***C***, we construct a gene based statistical test to measure the strength and significance of the association between genes and phenotype across experiments from different platforms and levels. For the *i*-th gene *G*_*i*_, $\left\{{\hat{C}}_{i}^{\;(j)}|j=1,\;2,\ldots ,\;T\right\}$ indicates the corresponding coefficient vector estimated from the *j*-th experiment, denoted by $\left[{\hat{C}}_{i,\;1}^{\;(j)},{\hat{C}}_{i,\;2}^{\;(j)},\ldots ,{\hat{C}}_{i,\;m_{i}}^{\;(j)}\right]$, where *m*_*i*_ is the number of features annotated to gene G_i_ in the *j*-th experimental dataset. The null hypothesis is there is no association between the *i*-th gene and phenotype in all *T* experiments, denoted by $H_{0}:\;{\left[{\hat{C}}_{i}^{\left(1\right)}{}^{\prime },{\hat{C}}_{i}^{\left(2\right)}{}^{\prime },\ldots ,{\hat{C}}_{i}^{\left(T\right)}{}^{\prime }\right]}^{\prime }=0$, vs. the alternative hypothesis *H*_*A*_: ${\hat{C}}_{i}^{\left(k\right)}$ ≠ 0, *k* = 1, 2, …, *T* for some *k*. To test the hypothesis, we summarize the coefficients of the *i*-th gene on all datasets as follows.

(7) $${\hat{S}}_{i}\;=\;\sqrt{\sum_{j\;=\;1}^{T}{\left\|{\hat{C}}_{i}^{\;(j)}\right\|}_{2}^{2}}$$

where $\hat{S}$_*i*_, *i* = 1, 2, …, *Q* is the statistical value on all *Q* genes. Due to different number of features included in different genes, an adjustment for gene size is necessary. A permutation based approach is used to reduce the potential bias due to varying gene size. The standardized gene level statistic is given by

(8) $${\tilde{S}}_{i}=\frac{{\hat{S}}_{i}-{\hat{S}}_{i}^{0}}{{\hat{\sigma }}_{i}}$$

where $\hat{S}$^0^_*i*_ and $\hat{\sigma }$_*i*_ are the mean and standard deviation of the *i*-th gene under the null hypothesis. Samples are permuted B times to construct null distribution of $\hat{S}$_*i*_, denoted by $\hat{\Gamma }$^0^_*i*_ = {$\hat{S}$^0^_*i*,*j*_|*j* = 1, 2, …, *B*}. $\hat{S}$^0^_*i*_ and $\hat{\sigma }$_*i*_ are then estimated based on permutation data. Since all $\hat{S}$^0^_*i*,*j*_ have been normalized, we could pool all $\hat{\Gamma }$^0^_*i*_ into a set Γ^0^ = {$\hat{\Gamma }$^0^_*i*_|*i* = 1, 2, …, *Q*} as the estimated null distribution. Therefore, the gene-level *p*-value of the *i*-th gene can be calculated by

(9) $$p_{i}\;=\;\frac{\#\;of\;\{\Gamma^{0}\geq {\hat{S}}_{i}\}}{\#\;of\;\{\Gamma^{0}\}}$$

### Simulation

To evaluate the performance of our proposed integrative method for identifying biomarkers, we simulated two levels of measurements: SNP and gene expression, and assigned different sample size for each dataset.

For each simulation, we generated 3 SNP datasets and 3 gene expression datasets. The sample sizes were 600, 400, and 200 for SNP data and 70, 50, and 30 for gene expression, respectively. 200 genes were simulated in each dataset. To mimic the linkage disequilibrium (LD) structure among SNPs, we chose a chromosome, chromosome 22, from HapMap CEU panel with phase III data and sample subjects by software HAPGEN2 (Su et al., 2011). Those SNPs were kept after the following filters were applied: (1) Minor allele frequency (MAF) at least 5%; and (2) Hardy-Weinberg Equilibrium (HWE) with significant level less than 0.001. We generated a dataset consisting of 15,235 SNPs which were assigned to 576 genes as the gene pool. Assuming an additive genetic model, each SNP was recorded as the count of minor allele (denoted as A) at that locus and thereby was valued by 0 (homozygote of major allele, aa), 1 (heterozygote, Aa) and 2 (homozygote of minor allele, AA). 200 genes including more than 10 SNPs were randomly selected from the pool, of which 20 genes were chosen as causal genes and 2 SNPs with MAF from uniform distribution (Unif) (0.15, 0.25) from each causal gene were further used to induce causal genetic effects on gene expression. The number of SNPs from 200 selected genes was randomly set from Unif(10,100) and those non-causal SNPs in each gene were selected from pooled SNPs.

We used SNP data to generate gene expression and phenotype data, referring to the similar method in Huang et al. (2014). Three SNP datasets with 70, 50, and 30 subjects were first simulated, as described in the method section. For each causal gene, e.g., gene *i*, the expression value *G*_*i*_ was derived from the causal SNPs in this gene by

(10) $$G_{i}=\sum_{j\;=\;1}^{n}SNP_{causal}^{\;j}\beta_{j}+\varepsilon$$

where n was the number of causal SNPs included in *G*_*i*_; and β_*j*_ indicated the effect of the *j*-th causal SNP(*SNP*^*j*^_*causal*_) on *G*_*i*_. We set β value from Unif(1, 1.2) and noise ε from normal distribution *N*(0, 1). The other non-causal gene expression values were generated by multivariate normal distribution *N*(0, Σ), where Σ was the covariance matrix of gene expressions, and the expressions of gene *i* and *j* have correlation coefficient 0.3^|*i* − *j*|^. Based on the simulated gene expression, the phenotype was generated by the following formula:

(11) $$logit\{Pr(Y_{i}=1)\}=\sum_{j\;=\;1}^{m}G_{causal}^{\;j}\tau_{j}+\varepsilon^{\prime }$$

where m was the number of causal genes, i.e., *m* = 20 in this study; *G*^*j*^_*causal*_ was gene expression for the *j*-th causal gene and τ_*j*_ was the corresponding effects on the outcome. The logit function was used to generate binary outcome. The identity function can be used if the quantitative phenotype was used. ε′ was non-genetic variable, which was assumed to follow normal distribution *N*(0, 1).

## Results

### Synthetic data

We assessed the performance of the two proposed sparse multitask models- multitask sglasso and multitask sgridge-on each single dataset and all datasets, respectively, and also compared them with widely used meta-analysis on three SNP datasets (meta-SNP) and three gene expression datasets (meta-EXP). Meta-analysis was implemented by the software MetaL (Willer et al., 2010).

#### Simulation 1: Fixed effect of causal genes in diverse dataset

In this simulation, we studied the scenario that the effects of causal genes across diverse datasets were fixed, i.e., τ^1^_*j*_ = τ^2^_*j*_ = … = τ^6^_*j*_, *i* = 1, 2, …, *m*, which indicated a causal gene had the same effect on all datasets. For *m* casual genes, first, we set a baseline vector η ∈ *R*^*m*^ from Unif(0.2, 2) and Unif(−2, −0.2). Next, to evaluate the performance of different methods on identifying casual genes under different levels of effects, a factor δ = 0, 0.2, 0.4, 0.6, 0.8, 1.0 was multiplied by η to have the final value of gene effects τ = η × δ. 50 replicates were performed and *B* = 500 permutations in each replicate were implemented to calculate empirical *p*-value of sparse multitask models. Finally, we compared the results of the following eight cases: multitask-sglasso on three expression datasets, three SNP datasets, and all six datasets; multitask-sgridge on three expression datasets, three SNP datasets and all six datasets; meta-analysis on three SNP datasets and three expression datasets.

**Figure 1:**
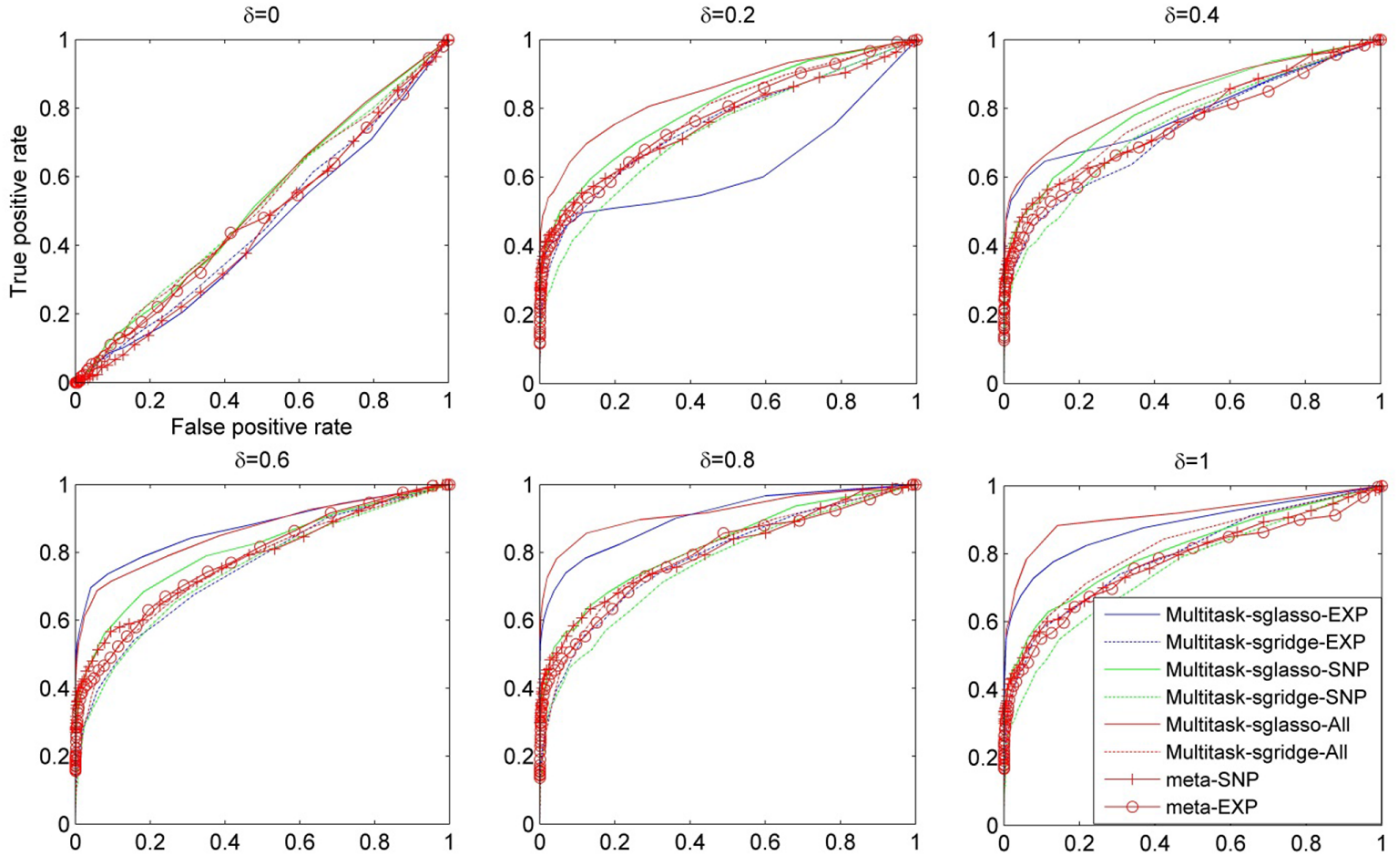
**The ROC curves for the comparison of eight cases: sparse multitask-sglasso and multitask-sgridge methods on three SNP datasets, expression datasets and all datasets, and meta-analysis on SNP and expression datasets, respectively**.

Figure 1 shows the comparison result of a set of methods under different values of δ, i.e., [0, 0.2, 0.4, 0.6, 0.8, 1.0]. The ROC curves were plotted using the false positive rate against true positive rate by varying the *p*-value threshold from 10^−4^ to 1. It could be seen that all methods had similar performance when there were no effective causal genes in all datasets (i.e., δ = 0). When the effects of causal genes (i.e., δ) increase, i.e., more variability of phenotypes could be explained by genetic variants, multitask-sglasso method shows better performance by removing the irrelevant genes with improved signal to noise ratio. When δ was greater than 0.2, multitask-sglasso methods on SNP, expression and both datasets significantly outperformed the other methods. This indicates that Multitask-sglasso method showed better performance by integrating all datasets than that of using only one level of data. In addition, when δ was greater than 0.4, multitask-sglasso method using only SNP or expression datasets still gave higher power than meta-analysis method. Multitask-sgridge method had less power than multitask-sglasso method and only showed better performance than meta-analysis method when causal genes have high effect sizes.

#### Simulation 2: Dynamic effects of causal genes in diverse datasets

In this simulation, we consider the situation that a causal gene has different effects at different levels and platforms. This is more likely to happen for real datasets since multiple datasets are usually generated from different studies with different study protocols, profiling techniques, and experimental platforms, leading to dynamic effect sizes of casual genes. We aimed to compare the performance of our sparse multitask methods with meta-analysis for biomarker identification in this dynamic case. Six datasets were generated with the same sample size and causal genes as those in the first simulation study. We simulated the dynamic effects of causal genes at different datasets by setting τ_*j*_ ~ *N* (η, σ^2^), *i* = 1, 2, …, 6, where η was fixed effect as described above, and σ was standard deviation indicating the dynamic effect of genes across datasets. We changed the value of σ from 0 to 1 with the interval of 0.2 to show different extent of heterogeneity of causal genes across diverse datasets. 50 replicates were averaged to draw the ROC curve for comparison.

**Figure 2:**
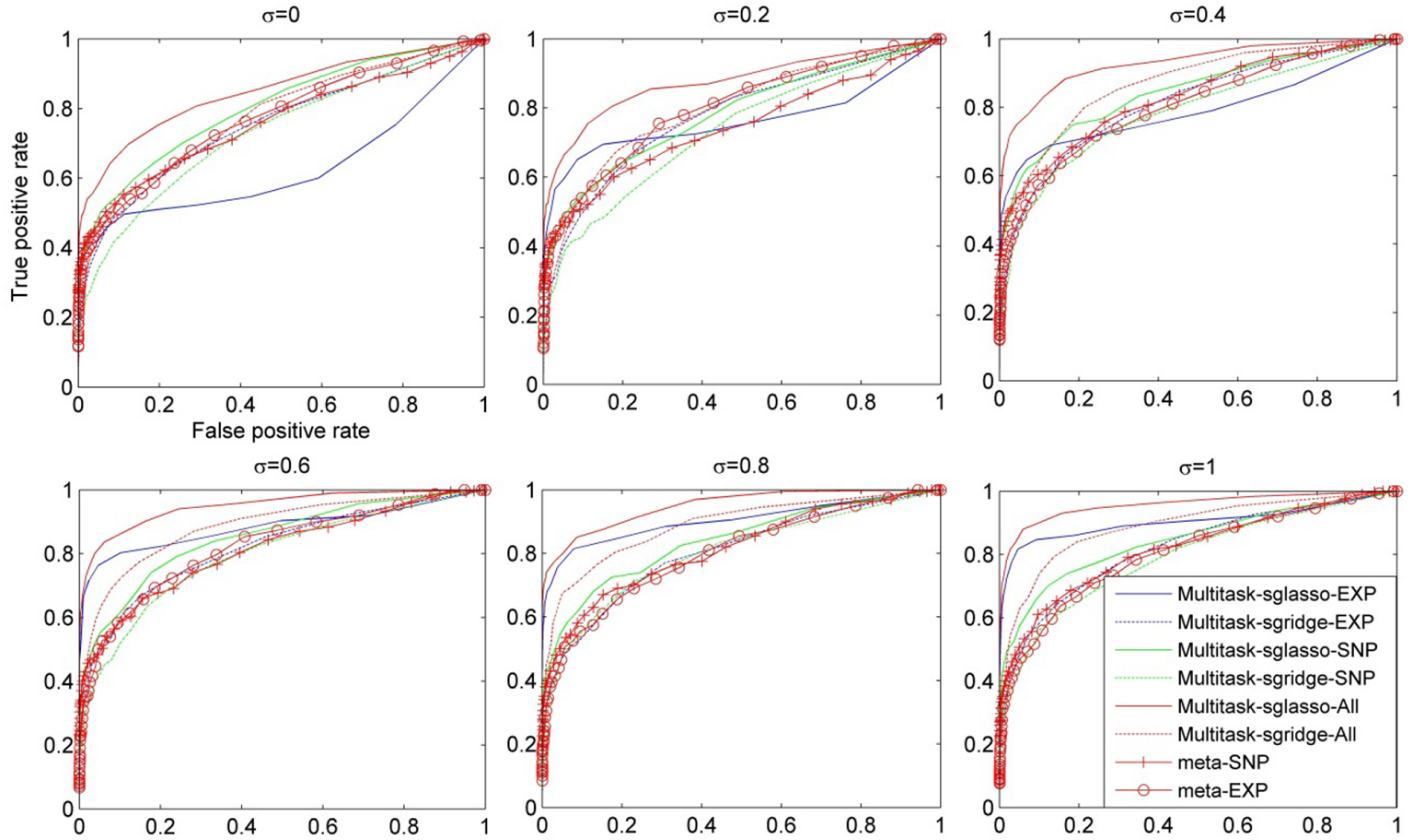
**The comparison of eight methods on three SNP and three expression datasets simulated with the dynamic model**. The variance of effect size of causal genes is set to normal distribution with variance varying from 0 to 1 at an interval of 0.2.

Figure 2 showed the comparison result of eight cases under dynamic effect models with variance of causal genes varying from 0 to 1. When σ = 0, the models reduced to the ones with fixed effects. When σ was greater than 0.4, sparse multitask-sglasso method on SNP, expression and both datasets significantly outperformed other methods in identifying casual genes. Except for sparse multitask-sglasso method, we can also see that the performance of sparse multitask-sgridge on all datasets was better than meta-analysis methods, which indicated the advantage of multitask method for integrating diverse datasets.

### Real data analysis

In this study, we took advantage of 3 gene expression datasets and 1 GWAS dataset with bone mineral density (BMD) measurements from our previous studies. The cohort I of gene expression data contained 80 Caucasian females, including 40 high and 40 low hip subjects (Chen et al., 2010b). The cohort II of gene expression data contained 19 Caucasian females, including 10 high and 9 low hip BMD subjects (Liu et al., 2005). The cohort III of gene expression data contained 26 Chinese females, all premenopausal and including 14 high and 12 low hip BMD subjects (Lei et al., 2009). For the GWAS dataset, SNP data were obtained using Affymetrix 500K arrays on 1,000 unrelated homogeneous Caucasians. After a suite of quality control procedures were performed, the SNP set for subsequent analysis contained 379,319 SNPs, yielding an average marker spacing of ~7.9 kb throughout the human genome (Xiong et al., 2009).

We combined gene expression and SNP datasets to identify those risk genes of BMD by our sparse multitask-sglasso integrative method. We chose one chromosome 6 containing the largest number of genes to perform gene-based analysis. 504 genes were included in the chromosome. More details in each dataset were given in Table 2.

**Table 2:** **A summary of four datasets from different levels and platforms used in this analysis**.

**Data type**	**Platform**	**Gene**	**Genetic variants**	**Sample**
SNP	Affymetrix 500K	504	10685	1000
Gene expression	HG-U133A	504	874	19
Gene expression	HG-U133A	504	1225	26
Gene expression	HG-U133A-Plus_2.0	504	874	80

**Figure 3:**
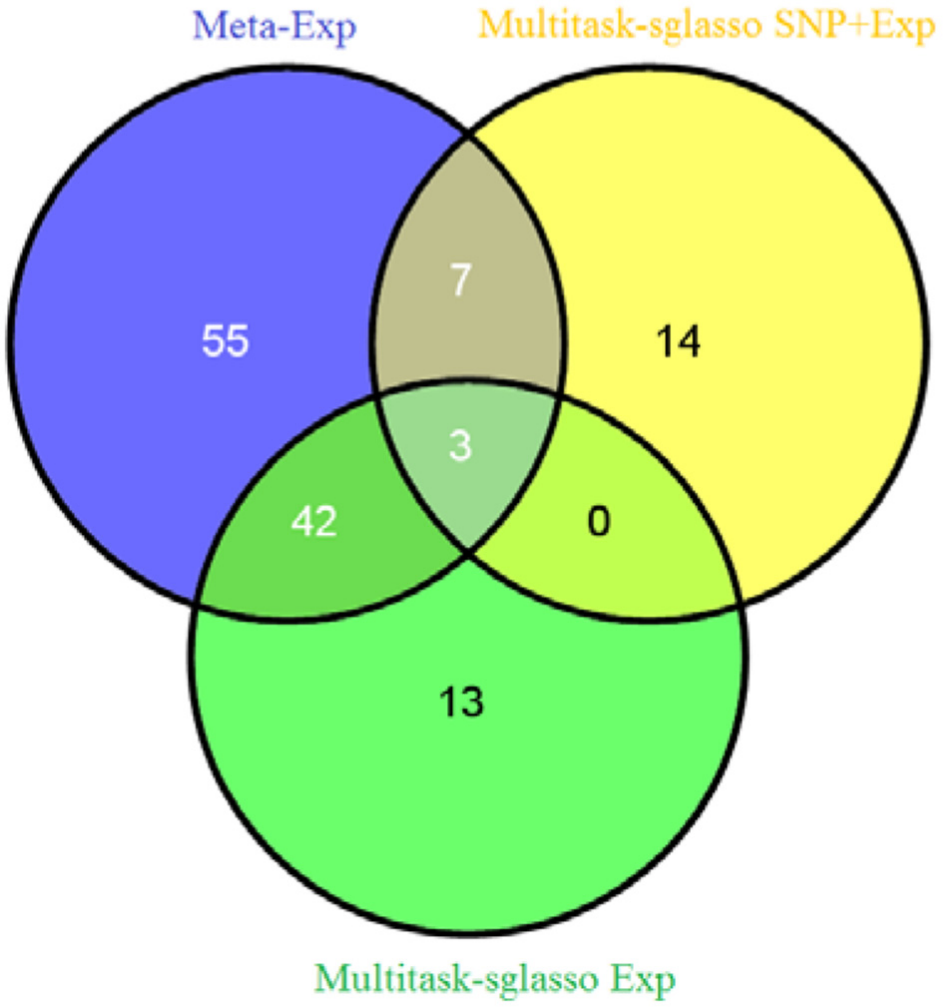
**The Venn diagram of identified genes by three methods: meta-analysis on EXP-19 and EXP-80 datasets, multitask-sglasso on all three expression datasets and multitask-sglasso on all gene expression and SNP datasets**.

We applied sparse multitask-sglasso method to SNP, gene expression and both datasets, respectively. To compare with meta-analysis, two gene expression datasets with the same level and experimental platforms, EXP-19 and EXP-80, were used for meta-analysis, denoted by meta-Exp. The most significant expression measurement in each gene was chosen to represent significance level of the gene. Figure 3 shows the Venn diagram of gene list by three methods: multitask-sglasso on all gene expression datasets, multitask-sglasso on all gene expression and SNP datasets, and meta-analysis on two expression datasets under the significant threshold 0.05. We could see that there were 45 genes shared by meta-Exp and multitask-sglasso on three expression datasets; 10 genes overlapped by meta-Exp and multitask-sglasso on both SNP and expression datasets; and three genes (“GPR116,” “HLA-DMB,” “PHACTR1”) identified by all methods. The small overlapping between multitask-sglasso Exp and multitask sglasso SNP + Exp is due to the use of additional information from large sample size of SNP dataset.

Table 3 lists 7 top significant genes identified and sorted by their *p*-values from sparse multitask-sglasso method on all datasets and the corresponding *p*-values by meta-analysis. Note that the *p*-values of the same gene usually were different in different studies. For example, SOD2 had much lower *p*-values in SNP and EXP-26 datasets than those in other datasets. This difference showed the dynamic effects of genes across diverse datasets with different levels and platforms. There are three genes (“TREML2,” “ HTR1E,” and “GLO1”) shared by sparse multitask-sglasso method on all of datasets and meta-Exp. Except for gene TREML2, the *p*-values of genes derived from all datasets were lower than those from the other methods, indicating higher level of significance given by our multitask method. The relatively smaller *p*-values of these genes in SNP data were due to the large sample size of SNP dataset, which will give more confidence on the findings.

**Table 3:**
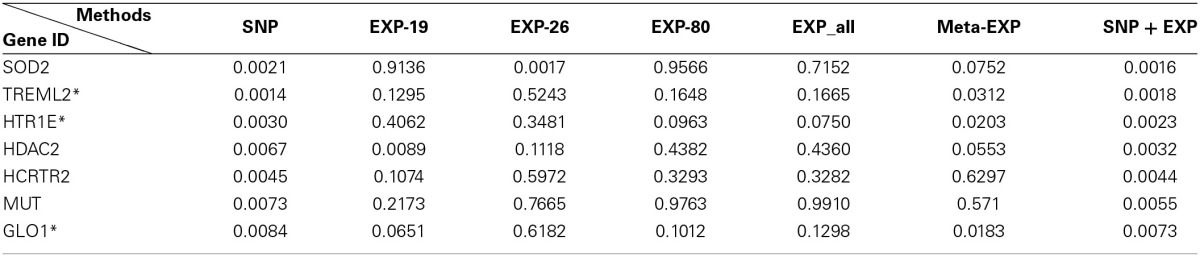
**The top 7 identified genes and their *p*-values by sparse multitask-sglasso method in bone mineral density studies**.

^*^ Genes identified by both meta-Exp and sparse multitask-sglasso on all datasets.

To further evaluate the significance of identified genes by multitask-sglasso, we performed gene level meta-analysis on three independent BMD studies for validation, more details were shown in supplementary data. The result (Table S1) listed the *p*-values of 24 identified genes based on single studies and meta-analysis. Most of these genes showed significant effects on BMD (*p* < 0.01), indicating the effectiveness of our sparse multitask regression method in identifying genetic risk factors.

Three shared genes (“TREML2,” “HTR1E,” and “GLO1”) may have important biological functions related to BMD associated with osteoporosis. TREML2 (also known as TLT-2) was located in a gene cluster on chromosome 6 with the single Ig variable (IgV) domain activating receptors TREM1 and TREM2, while these TREM receptor families were found to participate in the process of bone homeostasis by controlling the rate of osteoclastogenesis and regulating the differentiation of osteoclasts (Klesney-Tait et al., 2006; Otero et al., 2012). HTR1E was recently identified to contain SNPs significantly associated with a linear combination of multiple osteoporosis-related phenotypes including BMD (Karasik et al., 2012). GLO1, as a binding protein of methyl-gerfelin (M-GFN), was found to be able to result in the inhibition of osteoclastogenesis (Kawatani et al., 2008). Besides these three common genes, our method was also able to identify other osteoporosis-susceptible genes but was undetectable by meta-analysis. For instance, SOD2 has been identified as the gene susceptible to osteoporosis in our previous integrative analysis of mRNA, SNP, and protein data (Deng et al., 2011). It may play a significant role in BMD variation and pathogenesis of osteoporosis. HDAC2, as a member of histone deacetylases (HDACs), was found to play a critical role in bone development and biology (McGee-Lawrence and Westendorf, 2011). These genes were missed out with meta-analysis but can be detected with our proposed method, showing improved sensitivity.

## Conclusion and discussion

In this work, we proposed a multi-omics integration method, i.e., sparse group multitask regression model, which can integrate multiple genomic datasets from different levels, platforms, and subjects for gene based analysis. An efficient computational algorithm based on ADM was provided for its solution. The performance of the model was compared with meta-analysis in simulation datasets. The simulation results showed that our sparse group multitask regression model can increase the power of detecting risk genes by integrating multiple diverse datasets effectively. In particular, multitask-sglasso model outperformed meta-analysis method in simulations on genes with both fixed and dynamic effects. Our real data analysis on osteoporosis studies identified significant genes but missed by meta-analysis, and these genes were reported to be highly susceptible to BMD and osteoporosis. Overall, the advantages of our sparse group multitask regression method for biomarker identification from multiple omics datasets include: (1) it can combine diverse and complementary omic datasets without; (2) group the features by gene or gene set to account for the group structures in data (e.g., LD structure, co-expression, and genetic regulatory network); (3) remove irrelevant genes and/or features within a gene simultaneously.

Our proposed sparse multitask regression model provided a general framework for integrative analysis of diverse datasets. To fuse multiple diverse datasets, we considered the regression on each single dataset as a single task and then combined all single tasks into the model. Two sets of parameters were used in the model. ω_i_s were used to weight object functions (i.e., data fitting term at each level) different levels, while δ_*j*_ were used for different platforms. Similar to other works, we set ω to be equal by assuming each level of genetic data contains the same information (Ma et al., 2011). We assign δ_*j*_ to the data from different platforms by their sample sizes (Wilson and Lipsey, 2001). Other methods can also be applied to estimating weights such as Kaplan–Meier estimate (Liu et al., 2013) and inverse variance (Wilson and Lipsey, 2001). In order to account for the group effects and reduce the large number of features, we used two group sparse penalties in our multitask regression models, i.e., sparse group lasso and sparse group ridge, respectively. These penalties can perform feature selection at both group level and individual for multiple dataset levels, showing better performance than those of using lasso and group lasso penalties for single dataset analysis. Similar regression models were also recently proposed for using two-level sparse group penalties such as group bridge and group MCP (Huang et al., 2012). Ma et al. has recently applied these penalties in regression model for cancer studies to identify those risk oncology genes by integrating multiple expression level datasets from different cancer studies (Liu et al., 2013). Chen et al. has also compared and found that sparse group ridge outperformed group bridge penalty in single dataset regression model (Chen et al., 2010c). However, no study has been performed to compare them for multiple dataset integration and further work is needed in this direction.

## Web sources

The gene expression datasets from three cohorts can be accessed in GEO database (http://www.ncbi.nlm.nih.gov/geo/) with the following accession numbers: 19 Caucasians BMD study (GSE2208), 26 Chinese study (GSE7158), and 80 Caucasians study (GSE56815).

### Conflict of interest statement

The authors declare that the research was conducted in the absence of any commercial or financial relationships that could be construed as a potential conflict of interest.
