# Supplementary material for: Integrative analysis of multiple diverse omics datasets by sparse group multitask regression
Source: Front Cell Dev Biol. 2014 Oct 27;2:62. doi: 10.3389/fcell.2014.00062 (PMC4209817; doi:10.3389/fcell.2014.00062)
Supplement: Supplementary file 1 [file Table1.DOCX]

**Validation of identified genes by multitask-sglasso in three independent GWAS samples**

Three GWAS samples were used to exam the association of 24 identified genes on BMD by meta-analysis. Details of three GWAS were provided in [[1](#_ENREF_1)]. Two were from in-house studies and one was obtained from the database of genotype and phenotype (dbGAP). One of in-house samples consisted of 2250 unrelated individuals of European ancestry(CEU), and the other one had 1547 unrelated individuals of Chinese Han ancestry(CHI). The third sample was derived from the Framingham Heart Study (FHS), with 3747 phenotyped individuals of European ancestry. Individuals in each sample had hip BMD measured by dual-energy X-ray absorptiometry (DXA) scanners. Each GWAS sample was genotyped by high-throughput SNP genotyping array and was imputed by relevant population’s reference haplotypes in 1000 genomes project (1000G). SNPs with high accuracy in at least two samples and MAF > 0.05 in at least one sample were included in the association analysis. In unrelated GWAS samples, the linear regression model was used in MACH2QTL to examine the association between BMD and allele dosages as the predictor [[2](#_ENREF_2)]. In familial GWAS sample FHS, a mixed linear model was used in which the effect of genetic relatedness within each pedigree was also taken into account.

A SNP was assigned to a gene if it was located within or 50 kb upstream/downstream of the gene. Each gene was assigned a gene-wise *P* value using the *P* value of the gene's most significant SNP. Fisher's combination test was used to combine P-values of each gene in three GWAS samples. The result was shown in Table S1.

Table S1. p-values of 24 genes by independent test on three GWAS samples and meta-analysis

| Gene ID | p_CEU | p_CHI | p_FHS | score | p_meta |
| --- | --- | --- | --- | --- | --- |
| OR12D3 | 3.9E-03 | 2.3E-03 | 1.5E-05 | 4.5E+01 | 3.7E-08 |
| HLA-DMB | 7.5E-03 | 8.0E-03 | 5.0E-04 | 3.5E+01 | 5.0E-06 |
| OR10C1 | 2.6E-03 | 1.5E-02 | 1.6E-03 | 3.3E+01 | 9.9E-06 |
| PHACTR1 | 2.8E-03 | 1.9E-02 | 2.5E-03 | 3.2E+01 | 2.0E-05 |
| HLA-DPB1 | 6.2E-04 | 5.3E-02 | 4.7E-03 | 3.1E+01 | 2.2E-05 |
| IL20RA | 5.4E-03 | 3.4E-03 | 1.7E-02 | 3.0E+01 | 3.9E-05 |
| JARID2 | 8.5E-03 | 7.9E-03 | 5.2E-03 | 3.0E+01 | 4.4E-05 |
| GPR116 | 1.6E-02 | 5.8E-03 | 4.4E-03 | 2.9E+01 | 5.2E-05 |
| TREML2 | 1.7E-02 | 4.5E-03 | 1.2E-02 | 2.8E+01 | 1.0E-04 |
| GLO1 | 7.5E-03 | 3.0E-02 | 7.8E-03 | 2.6E+01 | 1.8E-04 |
| HDAC2 | 1.1E-02 | 4.8E-02 | 4.9E-03 | 2.6E+01 | 2.5E-04 |
| SEC63 | 9.7E-03 | 1.1E-02 | 3.1E-02 | 2.5E+01 | 3.1E-04 |
| TCP11 | 6.6E-03 | 2.1E-02 | 3.1E-02 | 2.5E+01 | 3.8E-04 |
| MUT | 8.5E-03 | 5.2E-03 | 1.3E-01 | 2.4E+01 | 4.9E-04 |
| HTR1E | 2.2E-03 | 4.3E-02 | 6.1E-02 | 2.4E+01 | 5.0E-04 |
| ETV7 | 1.3E-02 | 3.5E-02 | 2.2E-02 | 2.3E+01 | 8.2E-04 |
| PGK2 | 5.7E-03 | 1.0E-01 | 3.7E-02 | 2.2E+01 | 1.5E-03 |
| MTO1 | 3.2E-02 | 1.5E-02 | 4.8E-02 | 2.1E+01 | 1.5E-03 |
| HCRTR2 | 3.8E-02 | 9.2E-03 | 1.1E-01 | 2.0E+01 | 2.4E-03 |
| ALDH8A1 | 2.1E-02 | 9.2E-02 | 2.3E-02 | 2.0E+01 | 2.8E-03 |
| HIST1H2BB | 2.1E-02 | 6.7E-02 | 1.4E-01 | 1.7E+01 | 8.9E-03 |
| SOD2 | 7.6E-02 | 1.2E-01 | 2.7E-02 | 1.7E+01 | 1.0E-02 |
| APOBEC2 | 2.2E-01 | 7.8E-02 | 4.6E-02 | 1.4E+01 | 2.7E-02 |
| RNF146 | 9.8E-02 | 1.5E-01 | 1.2E-01 | 1.3E+01 | 4.6E-02 |

1. Zhang L, Choi HJ, Estrada K, Leo PJ, Li J, Pei Y-F, Zhang Y, Lin Y, Shen H, Liu Y-Z: **Multistage genome-wide association meta-analyses identified two new loci for bone mineral density**. *Hum Mol Genet* 2014, **23**(7):1923-1933.

2. Li Y, Willer CJ, Ding J, Scheet P, Abecasis GR: **MaCH: using sequence and genotype data to estimate haplotypes and unobserved genotypes**. *Genetic epidemiology* 2010, **34**(8):816-834.
